# Profile Trait Changes at Peak Height Velocity in Girls and Boys: A Longitudinal Study

**DOI:** 10.7759/cureus.90809

**Published:** 2025-08-23

**Authors:** Stela Kapoor, Roopal Patel, Dolly Patel, Vatsal Patel, Sanket Patel

**Affiliations:** 1 Orthodontics and Dentofacial Orthopedics, M.M. College of Dental Sciences and Research, Maharishi Markandeshwar (Deemed to be University), Ambala, IND; 2 Orthodontics and Dentofacial Orthopaedics, A.M.C. Dental College and Hospital, Ahmedabad, IND; 3 Orthodontics and Dentofacial Orthopaedics, K.M. Shah Dental College and Hospital, Vadodara, IND

**Keywords:** aesthetics, cephalometry, facial profile, growth and development, maturation, orthodontics, peak height velocity, pubertal peak, skeletal maturity, standing height

## Abstract

Objective

This longitudinal, correlational study aims to quantify skeletal and soft tissue facial changes in girls (9.5-10 years) and boys (12.5-13 years) during peak height velocity using lateral cephalograms. It further seeks to determine the strength and nature of associations between specific cephalometric parameters and incremental changes in standing height over defined time intervals.

Materials and methods

Forty subjects (girls: 9.5-10 years, boys: 12.5-13 years) were recruited through screenings. Lateral cephalograms and standing height measurements were recorded at three time points (T0, T1, T2). Height increments (T1-T0, T2-T1, T2-T0) were analysed. Dolphin 3D software v11.95 (Dolphin Imagine, Verona, Italy) assessed linear and angular cephalometric measurements.

Results

Height increased by 6.6 cm in girls and 6.9 cm in boys. In girls, standing height correlated significantly with B-Pg to Go-Me reduction (r=0.492, p=0.008, T2-T0), soft tissue chin thickness (r=0.511, p=0.030, T1-T0; r=0.492, p=0.038, T2-T0), and lower lip to chin length (p=0.016, T1-T0). In boys, standing height correlated with upper anterior facial height (r=0.501, p=0.034) and maxillary length (ANS-PNS) (r=0.553, p=0.017, T2-T0). ANB showed a moderate correlation with height (r=0.486, p=0.041, T2-T1), while lower lip to chin length correlated significantly (r=0.516, p=0.029, T1-T0).

Conclusion

Growth patterns in boys and girls are similar, but boys exhibit more pronounced increases. Significant correlations between cephalometric parameters and standing height were observed.

## Introduction

The phenomenon of facial growth unfolds as a meticulous and intricate process, characterized by gradual and distinct maturational stages from infancy to adulthood [1]. Craniofacial development involves three key aspects: growth, pattern, and rate. This intricate interplay involves a sequential and organized advancement of various facial components throughout an individual's lifespan. Growth varies in rhythm and magnitude despite its structured nature.

Longitudinal cephalometric studies show varied facial growth rates [2]. ‘Peak Height Velocity’ marks the fastest adolescent growth phase [1,3]. Maturity is assessed via age, dental stage, height growth, secondary sexual traits, and radiographic methods like cervical vertebral maturation Index (CVMI) and skeletal maturation index (SMI) [4]. Standing height changes strongly correlate with the pubertal growth spurt and serve as a simple, repeatable, non-invasive measure [5].

Orthodontic treatment is most effective before or during the pubertal growth spurt. Khadilkar et al. reported peak height velocities of 6.6 cm at 10.5 years in girls and 6.8 cm at 13.5 years in boys, establishing study age ranges of 9.5-10 years (girls) and 12.5-13 years (boys) [6].

Only a few longitudinal studies have examined facial profile changes during peak height velocity. Therefore, this longitudinal growth study aimed to study the growth changes in facial profile using lateral cephalogram of girls and boys aged 9.5-10 years and 12.5-13 years respectively, corresponding to the approximate ages of peak height velocity for girls (around 10.5 years) and boys (around 13.5 years); and to compare the observed changes between the time frames as well as between genders, using change in standing height as a parameter of reference.

## Materials and methods

This prospective, longitudinal study, approved by the Institutional Review Board (IRB) of the A.M.C. Dental College and Hospital (approval no. AMC/IRB/ORTHO/PG146/22), selected 40 subjects from 150 students via screenings, health checkups, dental camps, OPD visits, and pedodontia referrals.

The inclusion criteria included girls aged between 9.5 and 10 years and boys aged between 12.5 and 13 years at the commencement of the longitudinal study (T0) with a class 1 dental relationship, normal overjet and overbite with apparently average growth pattern. Subjects outside the specified age range, class II/III malocclusion, extreme horizontal/vertical growth pattern, prior orthodontic treatment, or syndromic conditions were excluded. 

Parents of eligible subjects, along with the subjects themselves, received verbal information about the study to obtain initial approval. Written assent and consent were obtained from patients and their guardians, respectively, who expressed interest in participation. Subjects were divided into two groups: Group 1 (20 girls) and Group 2 (20 boys). Upon their first arrival for recording data at the Department of Orthodontics and Dentofacial Orthopaedics, lateral cephalograms and height were recorded at T0.

**Figure 1:**
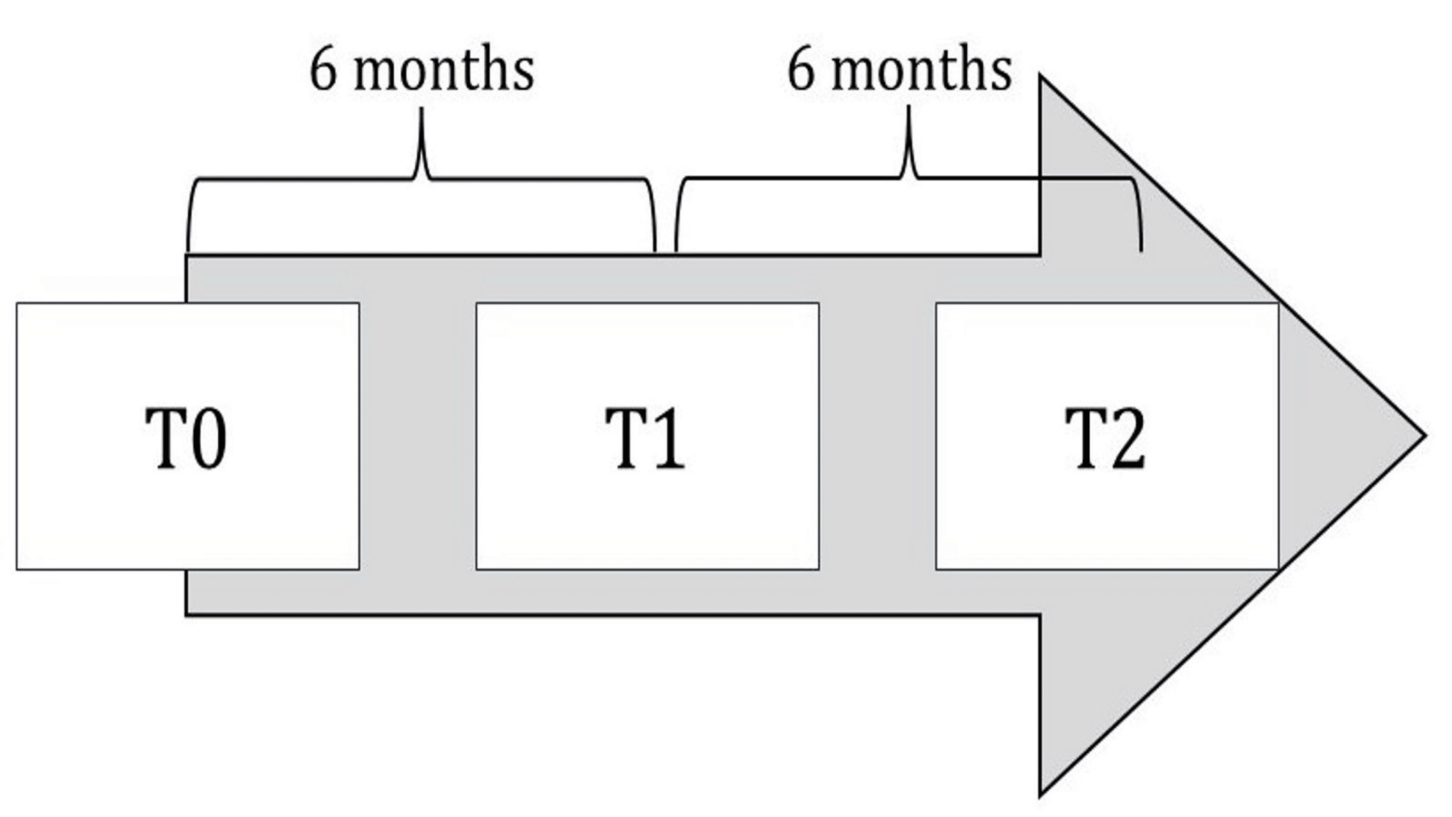
Time scale of the study is depicted, with T0 being the initial time point, T1 is 6 months after T0, T2 is 1 year after T0.

Procedures were repeated at T1 (6 months) and T2 (12 months). At T1, four subjects (2 girls, 2 boys) dropped out (within a 20% threshold), and none at T2 (Figure 1).

**Figure 2:**
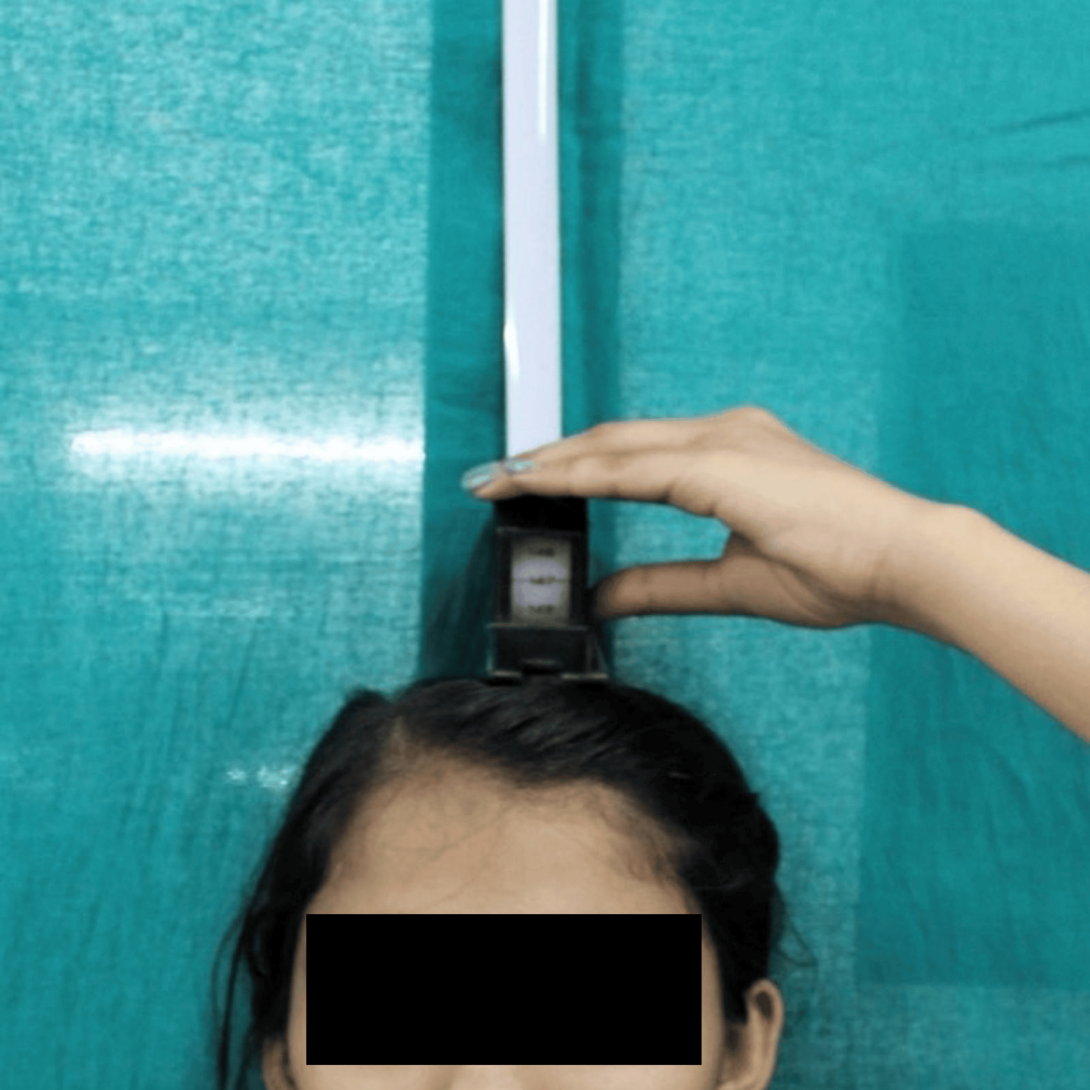
Height measurement using stadiometer.

Height was measured in natural head position (NHP), barefoot, during exhalation. A linear measurement was taken from the floor to the top of the head. In order to take the standing height measurement, the subject stood (without shoes) in their natural head position (parallel with the floor) and, whilst the subject breathed out, a linear measurement was made from the floor to the top of the subject’s head (Figure 2) [7].

**Figure 3:**
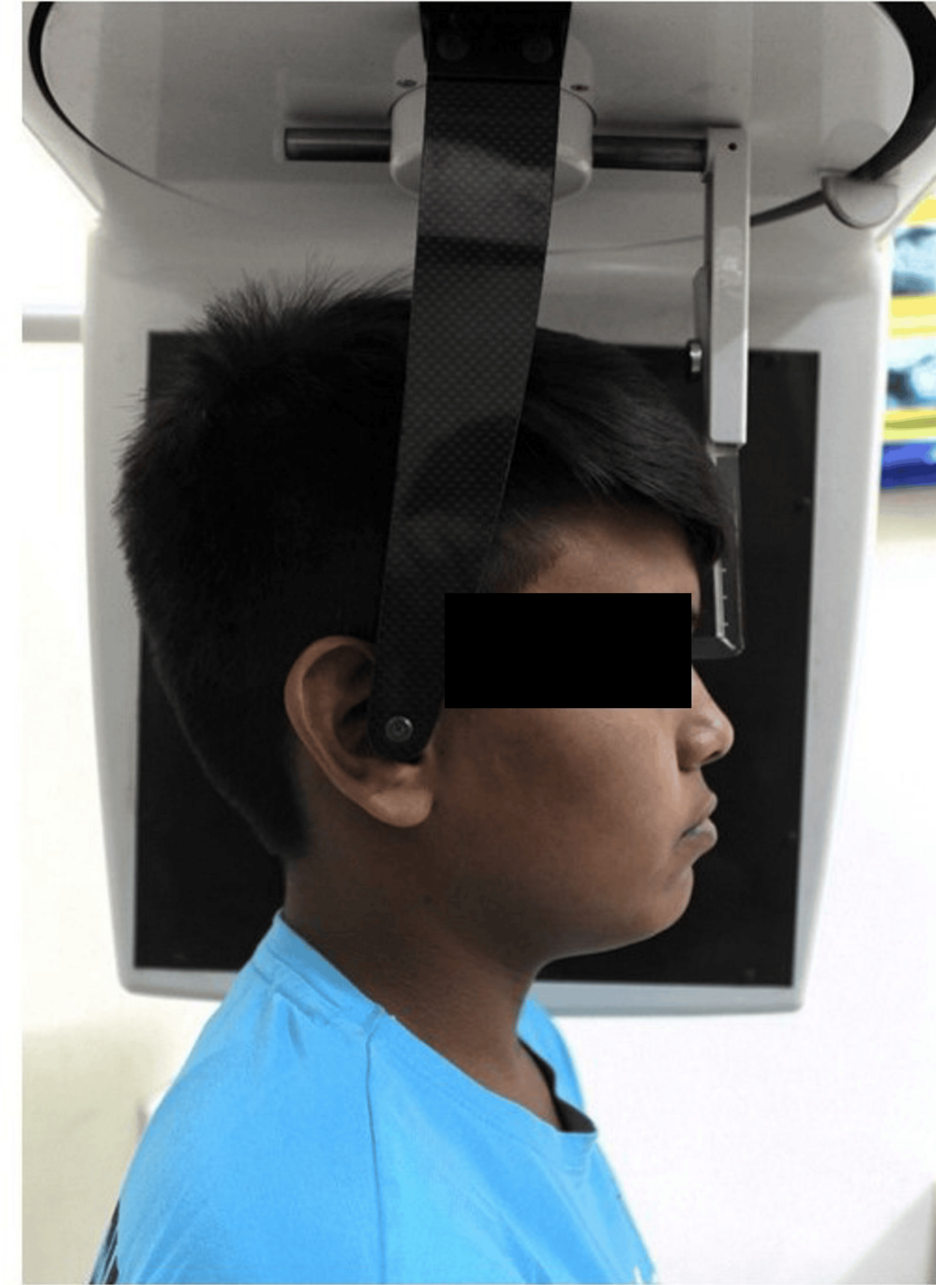
Subject in natural head position (NHP) for obtaining lateral cephalogram.

Cephalograms were taken using a Kodak 8000 Carestream digital system (Carestream Health, Rochester, USA). Technical specifications of the X-ray machine for cephalogram were as follows: tube voltage 72 kV, tube current 10 mA, exposure time 0.5 s. With the head in natural head position (NHP) [8,9] and using red laser light from the cephalostat as the guide, the cephalogram was obtained with maximum intercuspation and lips at rest (Figure 3).

**Figure 4:**
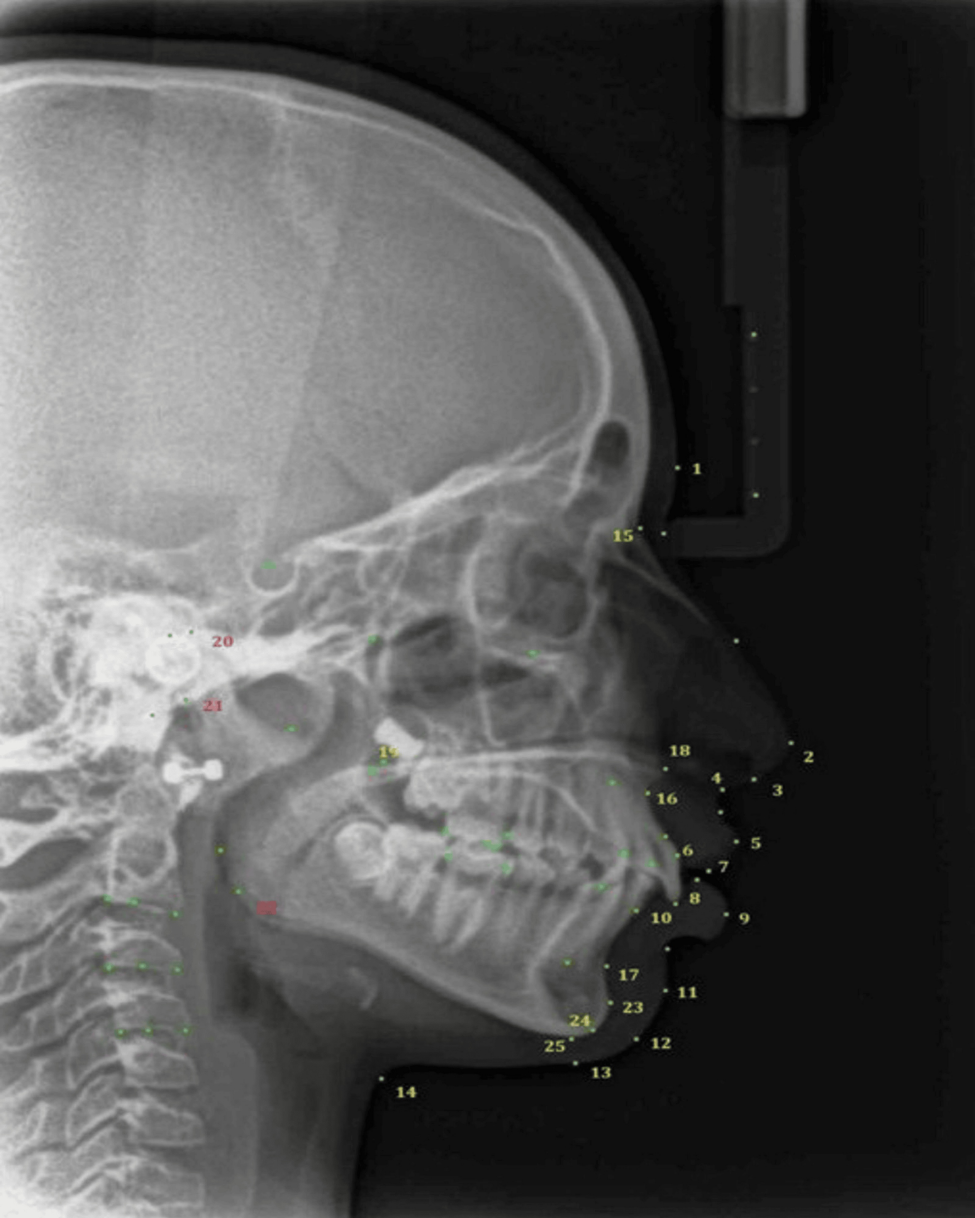
Cephalometric landmarks. Figure 4 shows the hard and soft tissue cephalometric landmarks used for customized analysis, with each numbered label identifying a specific anatomical point.

**Figure 5:**
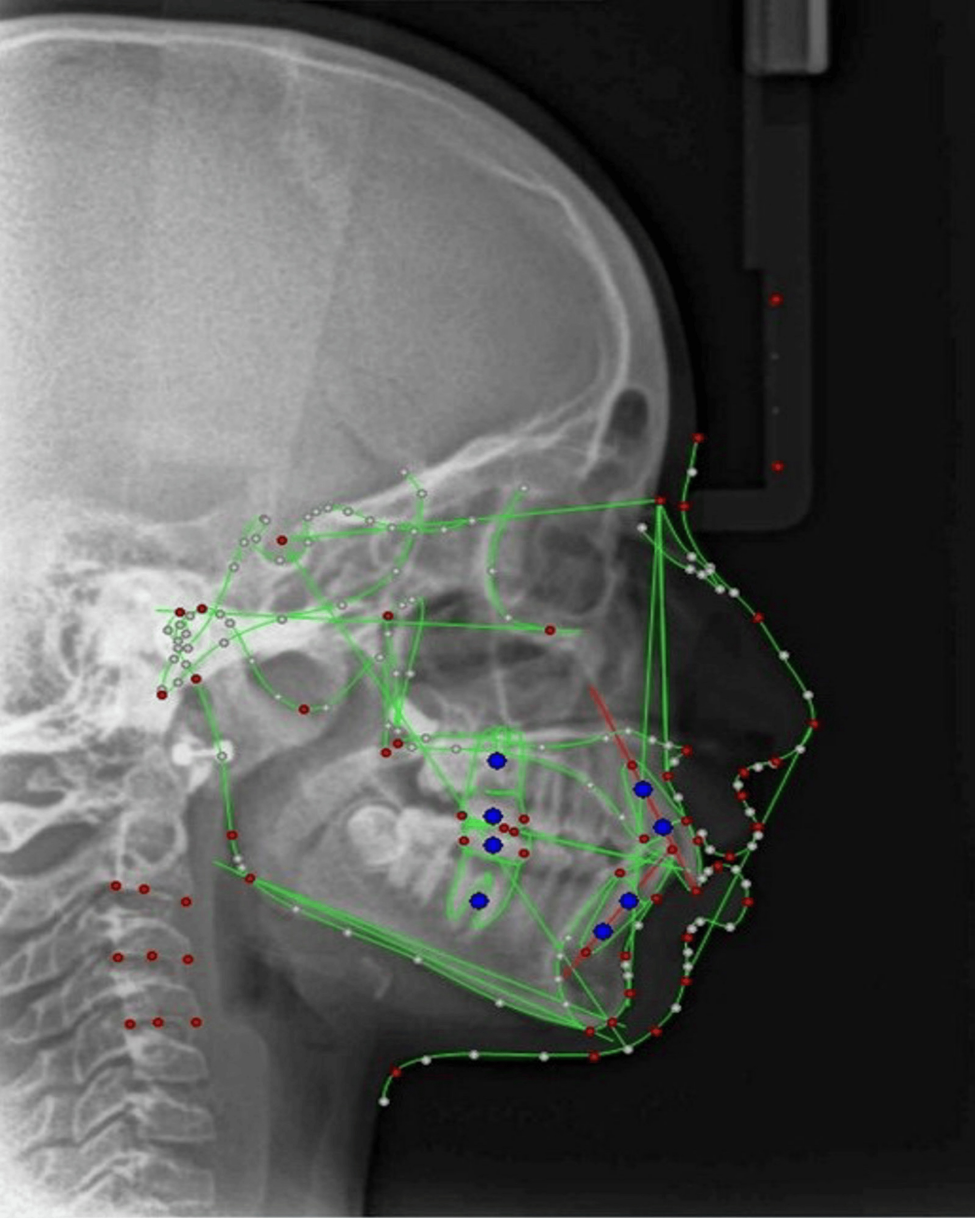
Digitization of lateral cephalogram using customized analysis. Figure 5 illustrates the digitization of a lateral cephalogram using customized analysis to measure predefined linear and angular cephalometric parameters.

Soft tissue traits from Bergman [10] were analysed for drape over hard tissues. Standing height and cephalometric parameters [11,12] were analyzed at T0, T1, and T2 (Table 1). A custom analysis comprising the soft and hard tissue landmarks was created in Dolphin 3D software using predefined soft and hard tissue measurements (Figure 4), both linear and angular (Figure 5). The obtained cephalograms were superimposed in Dolphin 3D using the Sella-Nasion line with Nasion as the registering landmark.

**Table 1:** Cephalometric parameters

Parameters	
A. Standing height (cm)	
B. Hard tissue parameters	
I. Sagittal skeletal relationship:	1. ANB (°)
II. Vertical skeletal relationships:	1. Upper anterior facial height (N-ANS) (mm)
	2. Lower anterior facial height (ANS-Me) (mm)
	3. GoGn to SN (Mandibular plane angle) (°)
III. Dimensional maxillary measurements:	1. Effective midfacial length (Co-A) (mm)
	2. ANS-PNS (Maxillary length) (mm)
IV. Morphologic and dimensional mandibular measurements:	1. Effective mandibular length (Co-Gn) (mm)
	2. Go-Pg (Mandibular corpus length) (mm)
	3. Gonial angle (Co-Go-Me) (°)
	4. B-Pg to Go-Me (°) (Symphyseal inclination)
C. Soft tissue parameters	
1. Nasal projection (Sn-P) (mm)	
2. Upper lip length (Sn-StS) (mm)	
3. Upper lip thickness (ULM-ULA) (mm)	
4. Lower lip to chin length (StI-Me’) (mm)	
5. Lower lip thickness (LLM-LLA) (mm)	
6. Soft tissue chin thickness (Pg-Pg’) (mm)	
7. Facial profile angle (G’-Sn-Pg’) (°)	
8. Nasolabial angle (Col-Sn-ULA) (°)	
9. Lower face-throat angle (Sn-Pg’-CP) (°)	

Statistical analysis

The descriptive statistics of cephalometric hard and soft tissue cephalometric measurements, along with standing height, were described separately for girls and boys. The comparison of change in cephalometric hard and soft tissue measurements along with standing height between girls and boys at three time frames, namely T1-T0, T2-T1, and total time frame T2-T0, was done using unpaired t-test to elucidate any gender-specific differences. In order to correlate the change in cephalometric hard and soft tissue cephalometric measurements and the incremental increase in standing height, Pearson’s correlation test was used for girls and boys separately.

## Results

Data was recorded in Microsoft Excel 2010 (Microsoft Corporation, Redmond, USA). The data on continuous variables is presented as mean ± standard deviation (SD). Statistical analysis was conducted using SPSS version 20.0 (IBM Corp., Armonk, USA). Statistical significance was set at P<0.001.

Table 2 shows the unpaired t-test for comparison of facial measurements of girls vs boys at T1-T0, T2-T1, and T2-T0 time frames.

**Table 2:** Descriptive statistics of measurements of girls (n=18) and boys (n=18)

Measurement	Values for Girls						Values for Boys					
	T0		T1		T2		T0		T1		T2	
	Mean	SD	Mean	SD	Mean	SD	Mean	SD	Mean	SD	Mean	SD
Standing Height (Cm)	133.7	9.2	136.8	8.6	140.3	8.7	140	10.3	143.3	10.5	146.9	10.5
ANB (°)	4	2	3.8	2.2	3.5	2.1	4.2	1.9	4	1.7	3.8	2
Upper Ant. Facial Height (mm)	43.8	2.7	44.5	2.6	45.6	2.6	44	3.1	46.1	3	46.9	2.4
Lower Ant Facial Height (mm)	50.4	3.3	51.2	2.9	52.1	3	55	4.5	56.4	4.5	57.4	4.9
GoGn-Sn (°)	29.5	5.7	29.5	6	28.9	6.3	29.7	3.7	29.6	3.4	29.4	3.3
Effective Midfacial Length(mm)	73.5	4.1	76	4.6	76.5	5.2	77.1	4.3	79.7	3.7	81.9	3.9
ANS-PNS (Maxillary Length) (mm)	47.3	3	48.8	2.9	50.1	2.9	48	2.2	49.8	1.9	51.1	2.1
Effective Mandibular Length (mm)	92.6	4.9	94.7	4.8	96.6	6.2	97.7	5.8	100.9	4.9	103.8	6.1
Go-Pg (mm)	63.1	4.5	65	5	67.4	5.3	66.1	5.4	68.4	3.6	70.4	3.8
Gonial Angle (°)	122.9	3.9	121.6	4	120.7	4.9	126.1	7.5	125.3	6	124.6	6.2
B-Pg To Go-Me (°)	60.3	8.2	58.9	7.8	58.2	7.6	58.6	5	57.4	6.3	56.7	7.1
Nasal Projection (mm)	10.8	1.4	11.4	1.8	11.8	1.8	10.3	1.1	11.5	2.6	11.5	1.3
Upper Lip Length (mm)	15.7	2	16.7	2.1	17.7	2.6	18.1	1.4	18.9	1.6	19.4	1.5
Upper Lip Thickness (mm)	9.2	1.5	10.3	1.7	10.8	1.5	10.1	2	10.6	1.8	11.3	1.5
Lower Lip To Chin Length (mm)	35.1	2.5	35.9	2.5	37.5	2.9	36.4	2.9	38.3	3.4	39.8	3.9
Lower Lip Thickness (mm)	9.8	1.3	10.3	1.3	11.2	1.3	11	2.4	11.5	2.4	12.3	2.3
Soft Tissue Chin Thickness (mm)	9.2	1.4	9.6	1.2	10	1.1	9.4	1.6	10	1.4	10.7	1.6
Facial Profile Angle (°)	159.1	6.7	157.5	7	157	7.2	157.9	6.6	156.8	6.3	156.4	6.6
Nasolabial Angle (°)	107.8	12.1	104.8	11.3	96.8	24.3	106.1	9.2	102.6	8	100.3	8.2
Lower Face-Throat Angle (°)	106	7.5	108.4	7.2	109.9	7.8	106	7	108.3	7.3	110.2	7.1

Boys had a greater increase in standing height than girls by 0.2 cm (T1-T0) and 0.1 cm (T2-T1), but the difference was not significant. The overall increase was greater in boys as compared to girls by 0.3 cm during T2-T0, but the difference was not statistically significant. A significant difference was observed in upper anterior facial height over T2-T0, where boys showed a greater increase than girls by 1.1 mm (p=0.026). A statistically significant difference was also noted during the initial time frame between time points T1 and T0 (T1-T0), during which boys displayed a greater increase in upper anterior facial height by 1.4 mm (p=0.035). Effective mandibular length showed a significant overall increase between the total frame time points T2 and T0 (i.e., T2-T0), during which boys displayed a greater increase in effective mandibular length by 2.1 mm (p=0.016).

Girls showed a significant increase in upper lip length during T2-T1 (p=0.043); however, the total increase over T2-T0 (0.7 mm) was not significant. Boys had a significant increase in lower lip to chin length by 1.1 mm (p=0.035) during T1-T0, though the total increase over T2-T0 was not significant. A statistically significant difference was noted in soft tissue chin thickness between time points T2 and T1 (T2-T1), during which boys displayed a greater increase in soft tissue chin thickness (p=0.035) and between time points T2 and T0 (T2-T0), during which boys displayed a greater increase in effective mandibular length by 0.4 mm (p=0.049).

Table 3 presents the correlation coefficients of standing height with other measurements across T1-T0, T2-T1, and T2-T0 for both genders. In girls, during the time frame T1-T0, the increase in lower anterior facial height was found to have a moderately positive correlation (r=0.554) with the increase in standing height, which is moderately significant (p=0.017). Effective mandibular length had a moderate positive correlation (r=0.593) with the increase in standing height during T1-T0, which is significant (p=0.010). The reduction in B-Pg to Go-Me angle had a strong positive correlation (r=0.746) with the increase in standing height during T1-T0, which is highly significant (p=0.000), and continued to be strongly positive (r=0.492) during the overall time frame (T2-T0), which is significant (p=0.008). The increase in lower lip to chin length had a moderate positive correlation during T1-T0, which is significant (p=0.016). Soft tissue chin thickness had a moderate positive correlation (r=0.511) with the increase in standing height during T1-T0, which is significant (p=0.030); the correlation continued to be moderate positive (r=0.492) with the increase in standing height during the overall time frame (T2-T0), which is significant (p=0.038).

**Table 3:** Unpaired t-test for facial measurements of girls vs boys - T1-T0, T2-T1, and T2-T0 *P<0.05: Moderately significant **P<0.001: Highly significant

Measurement	T1-T0						T2-T1						T2-T0					
	Girls		Boys		Mean Difference	P Value	Girls		Boys		Mean Difference	P Value	Girls		Boys		Mean Difference	P Value
																			Mean	SD	Mean	SD	Mean	SD	Mean	SD		Mean	SD	Mean	SD
	Standing Height (Cm)	3.1	3.3	3.3			1.2	-0.2	0.834	3.5		1	3.6	1.4	-0.1	0.766			6.6	4	6.9	2.1	-0.3	0.779
ANB (°)	-0.2	0.3	-0.2	0.4	0	1	-0.3	0.6	-0.2	0.5	-0.1	0.714	-0.5	0.5	-0.4	0.6	-0.1	0.442
Upper Ant. Facial Height (mm)	0.7	0.6	2.1	2.6	-1.4	0.035*	1.1	1	0.8	2.9	0.3	0.761	1.8	1	2.9	1.9	-1.1	0.026*
Lower Ant Facial Height (mm)	0.8	1.3	1.4	1.3	-0.6	0.157	0.9	0.6	1	0.7	-0.1	0.882	1.7	1.1	2.4	1.3	-0.7	0.142
GoGn-Sn (°)	0	1.6	-0.1	1.8	0.1	0.868	-0.6	0.8	-0.2	0.6	-0.4	0.07	-0.6	1.7	-0.3	2.3	-0.3	0.614
Effective Midfacial Length(mm)	2.5	3.8	2.6	2.6	-0.1	0.887	0.5	4.7	2.2	2.1	-1.7	0.194	3	2.3	4.8	3.3	-1.8	0.068
ANS-PNS (Maxillary Length) (mm)	1.5	1.5	1.8	1.5	-0.3	0.617	1.3	1.4	1.3	1.4	-0.1	0.789	2.8	1.5	3.1	1.8	-0.3	0.486
Effective Mandibular Length (mm)	2.1	1.3	3.2	1.8	-1.1	0.030*	1.9	2.3	2.9	2	-1	0.194	4	2.6	6.1	2.4	-2.1	0.016*
Go-Pg (mm)	1.9	1.2	2.3	3.1	-0.4	0.55	2.4	1.1	2	1.3	0.4	0.351	4.3	1.6	4.3	3.7	0	1
Gonial Angle (°)	-1.3	2.1	-0.8	2.2	-0.5	0.387	-0.9	2	-0.7	0.8	-0.2	0.783	-2.2	3.3	-1.5	2.6	-0.7	0.438
B-Pg To Go-Me (°)	-1.4	2.5	-1.2	2.2	0.2	0.686	-0.7	1.8	-0.7	1.4	0	1	-2.1	3.6	-1.9	3	-0.2	0.839
Nasal Projection (mm)	0.6	0.5	1.2	2.4	-0.6	0.304	0.4	0.4	0	2.3	0.4	0.704	1	0.6	1.2	0.9	-0.2	0.401
Upper Lip Length (mm)	1	0.8	0.8	0.6	0.2	0.627	1	0.8	0.5	0.6	0.5	0.043*	2	1.1	1.3	0.9	0.7	0.092
Upper Lip Thickness (mm)	1.1	0.9	0.5	1	0.6	0.054	0.5	0.5	0.7	0.5	-0.2	0.238	1.6	0.6	1.2	1.2	0.4	0.089
Lower Lip To Chin Length (mm)	0.8	1.6	1.9	1.4	-1.1	0.035*	1.6	1.2	1.5	1.1	0.1	0.796	2.4	2.2	3.4	2.3	-1	0.174
Lower Lip Thickness (mm)	0.5	0.7	0.5	0.4	0	1	0.9	0.5	0.8	0.4	0.1	0.312	1.4	1	1.3	0.6	0.1	0.593
Soft Tissue Chin Thickness (mm)	0.4	0.6	0.6	0.4	-0.2	0.599	0.4	0.3	0.7	0.6	-0.3	0.035*	0.8	0.6	1.3	0.6	-0.4	0.049*
Facial Profile Angle (°)	-1.6	2.7	-1.1	1.8	-0.5	0.543	-0.5	1	-0.4	0.9	-0.1	0.754	-2.1	3	-1.5	2.3	-0.6	0.527
Nasolabial Angle (°)	-3	4.6	-3.5	3.6	0.5	0.724	-8	22.8	-2.3	2.6	-5.7	0.3	-11	24.6	-5.8	4.5	-5.2	0.385
Lower Face- Throat Angle (°)	2.4	3.2	2.3	1.6	0.1	0.861	1.5	2.8	1.9	1.1	-0.4	0.564	3.9	5.7	4.2	2.3	-0.3	0.854
CVMI	0.4	0.5	0.1	0.4	0.3	0.278	0.2	0.4	0.5	0.5	-0.3	0.075	0.6	0.5	0.6	0.5	0	1

In boys, overall change in the upper anterior facial height during the total time frame (T2-T0), was found to have a moderately positive correlation (r=0.501) with the increase in standing height, which is moderately significant (p=0.034). Overall change in ANS-PNS (maxillary length) during the total time frame (T2-T0) was found to have a moderately positive correlation(r=0.553) with the increase in standing height, which is moderately significant (p=0.017). The correlation of change in ANB with the increase in standing height was moderately significant (r= 0.486) during T2-T1 (p=0.041). The increase in lower lip to chin length during T1-T0 had a moderate positive correlation (r=0.516), which is moderately significant (p=0.029).

**Figure 6:**
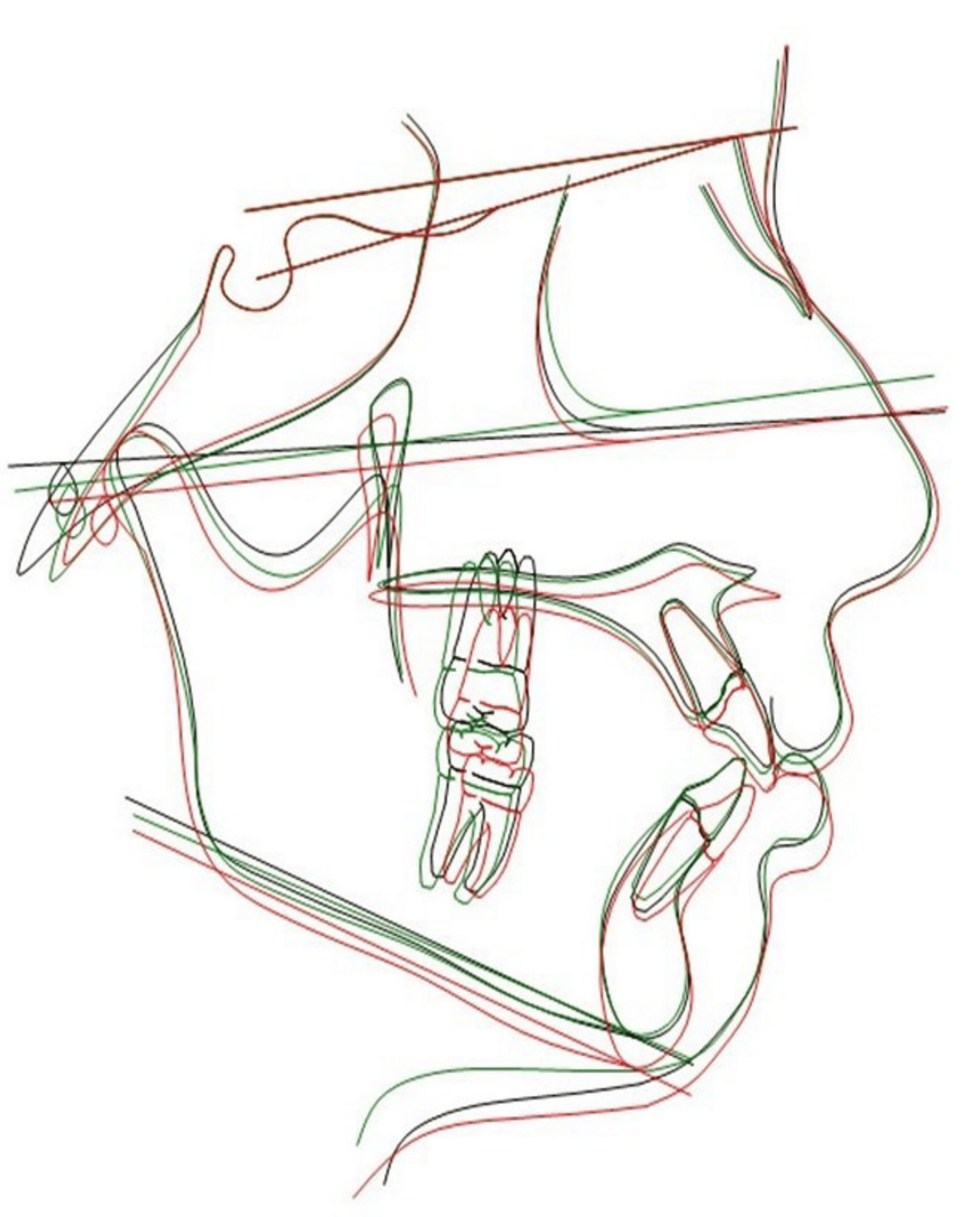
Superimposition of lateral cephalograms at T0, T1, and T2 in a girl Superimposition of lateral cephalograms of a girl obtained at time points T0, T1, and T2, on Dolphin 3D software v11.95 (Dolphin Imaging, Verona, Italy) using the Sella-Nasion line with Nasion as the registering landmark; T0 - black, T1 - green, T2 - red.

**Figure 7:**
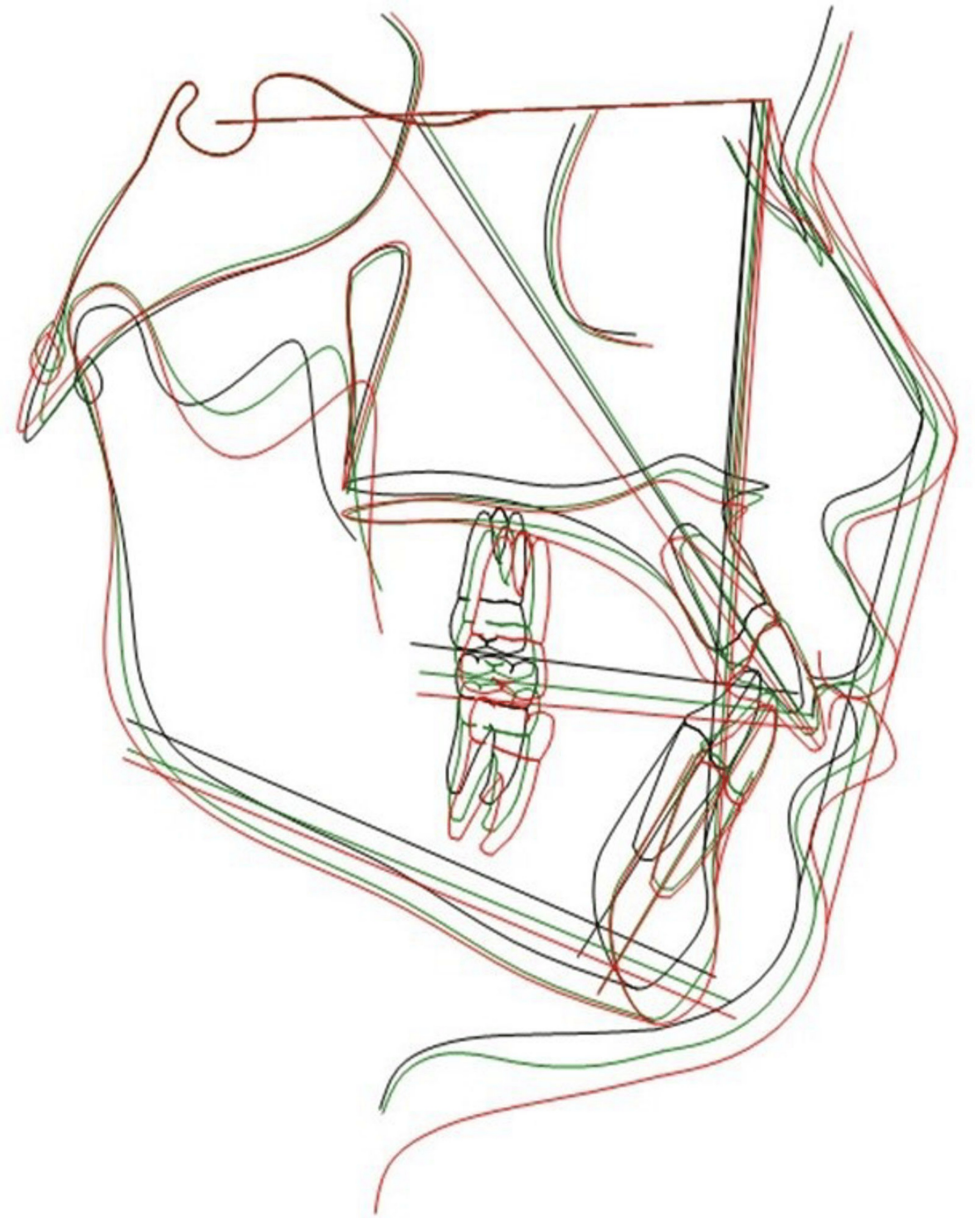
Superimposition of lateral cephalograms at T0, T1, and T2 in a boy Superimposition of lateral cephalograms at time points T0, T1, and T2 using Dolphin 3D software (version 11.95; Dolphin Imaging, Verona, Italy). The Sella–Nasion line with Nasion as the registering landmark was used; T0 - black, T1 - green, T2 - red

**Figure 8:**
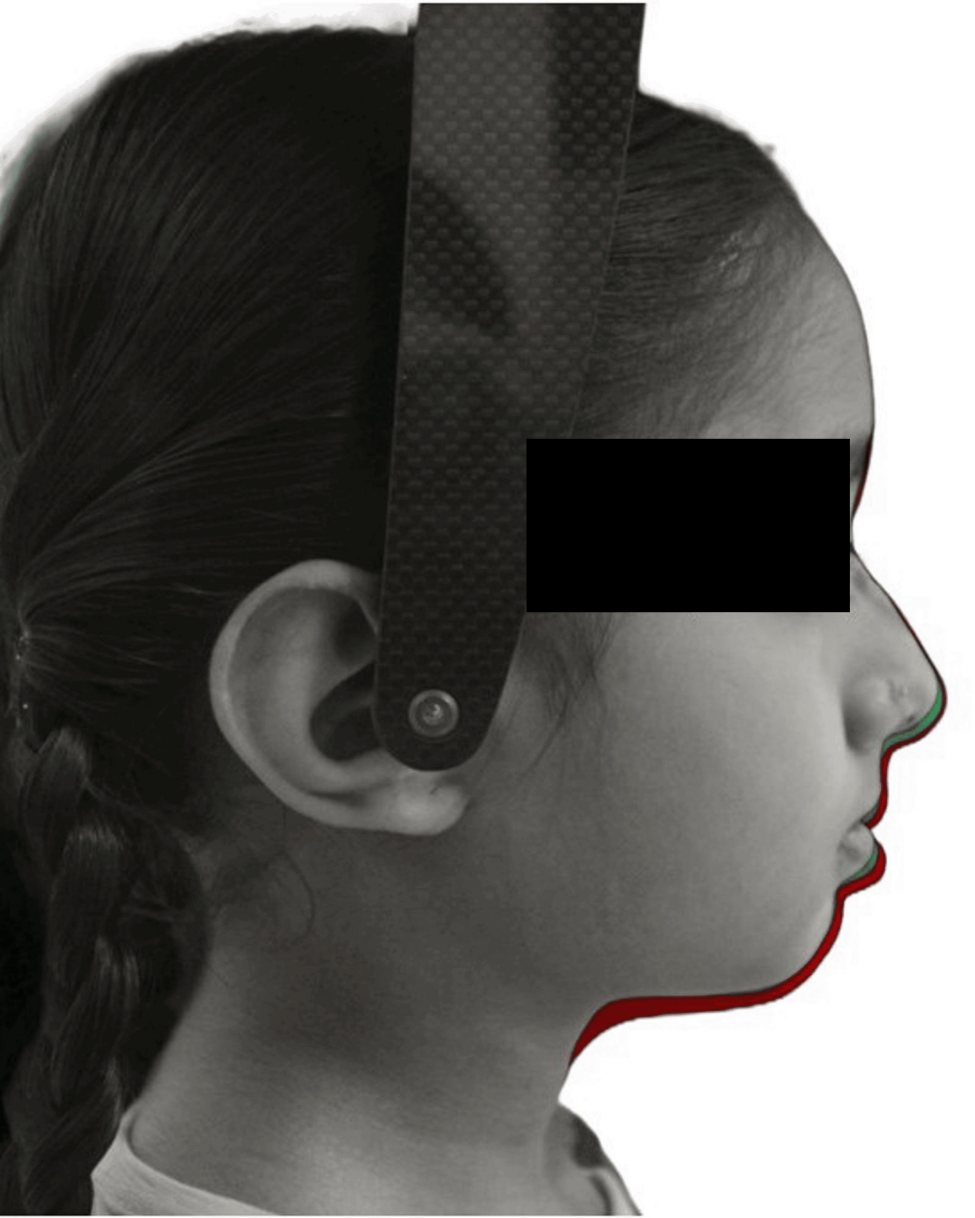
Photographic superimposition showing soft tissue changes from T0 (grey) to T1 (green) and T2 (red) in a girl.

**Figure 9:**
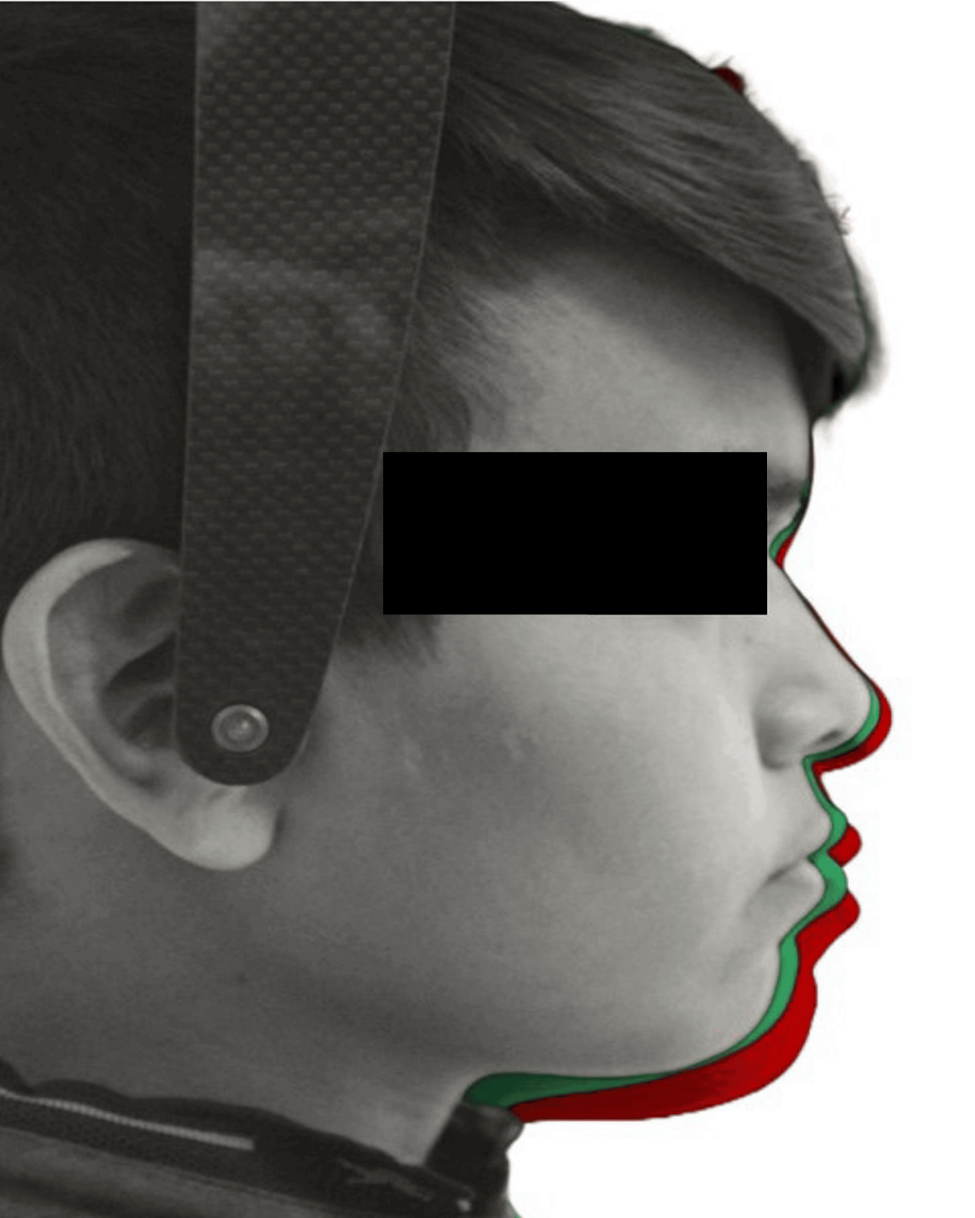
Photographic superimposition showing soft tissue changes from T0 (grey) to T1 (green) and T2 (red) in a boy.

The alterations in the hard tissue profile are distinctly evident upon superimposition of lateral cephalograms (Figures 6, 7), whereas soft tissue changes are best assessed through photographic superimposition (Figures 8, 9).

The correlation coefficient of standing height changes at T1-T0, T2-T1, and T2-T0, with other measurements at T1-T0, T2-T1, and T2-T0, respectively, among girls and boys is presented in Table 4.

**Table 4:** Correlation coefficient of standing height at T1-T0, T2-T1, and T2-T0 with other measurements at T1-T0, T2-T0, and T2-T0, respectively among girls and boys. *P<0.05: Moderately significant; **P<0.001: Highly significant.

Measurements	Value in Girls						Value in Boys					
	T1-T0		T2-T1		T2-T0		T1-T0		T2-T1		T2-T0	
	R	P Value	R	P Value	R	P Value	R	P Value	R	P Value	R	P Value
ANB (°)	-0.088	0.728	-0.303	0.222	-0.112	0.657	-0.092	0.716	0.486	0.041*	0.226	0.368
Upper Ant. Facial Height (mm)	0.088	0.729	-0.313	0.205	0.013	0.96	0.206	0.413	0.439	0.068	0.501	0.034*
Lower Ant Facial Height (mm)	0.554	0.017	-0.205	0.414	0.3	0.227	-0.028	0.914	0.338	0.17	0.236	0.345
GoGn-Sn (°)	-0.623	0.006*	0.355	0.149	-0.264	0.289	-0.229	0.36	-0.04	0.875	-0.005	0.984
Effective Midfacial Length(mm)	0.031	0.904	0.3	0.227	0.137	0.58	-0.115	0.649	0.189	0.452	0.102	0.686
ANS-PNS (Maxillary Length) (mm)	0.045	0.858	0.285	0.252	-0.062	0.806	0.086	0.735	0.262	0.293	0.553	0.017*
Effective Mandibular Length (mm)	0.593	0.010*	0.228	0.363	0.46	0.055	0.131	0.605	0.173	0.493	0.455	0.058
Go-Pg (mm)	-0.353	0.151	0.303	0.222	-0.111	0.661	-0.143	0.571	0.08	0.752	-0.201	0.424
Gonial Angle (°)	0.649	0.004*	-0.52	0.027*	0.255	0.306	-0.381	0.118	0.18	0.474	-0.29	0.243
B-Pg To Go-Me (°)	0.746	0.000**	0.241	0.336	0.607	0.008*	0.233	0.351	-0.479	0.044*	-0.132	0.601
Nasal Projection (mm)	0.178	0.481	-0.172	0.495	0.3	0.226	-0.338	0.17	-0.102	0.686	-0.213	0.397
Upper Lip Length (mm)	0.064	0.8	-0.168	0.505	0.057	0.822	-0.348	0.157	-0.078	0.758	-0.173	0.492
Upper Lip Thickness (mm)	0.157	0.534	-0.14	0.578	0.232	0.355	0.342	0.165	0.113	0.655	0.299	0.228
Lower Lip To Chin Length (mm)	0.558	0.016*	-0.051	0.84	0.4	0.1	0.516	0.029*	-0.201	0.423	0.289	0.245
Lower Lip Thickness (mm)	0.459	0.055	-0.309	0.212	0.077	0.76	-0.517	0.028*	-0.012	0.963	-0.254	0.309
Soft Tissue Chin Thickness (mm)	0.511	0.030*	0.163	0.518	0.492	0.038*	-0.33	0.18	0.369	0.131	0.268	0.282
Facial Profile Angle (°)	0.588	0.010*	-0.284	0.253	0.436	0.07	0.308	0.213	0.002	0.992	0.228	0.363
Nasolabial Angle (°)	-0.08	0.754	0.041	0.871	-0.142	0.575	-0.082	0.746	0.159	0.528	-0.165	0.514
Lower Face-Throat Angle (°)	-0.686	0.002*	-0.218	0.385	-0.535	0.022*	0.279	0.263	-0.034	0.893	0.292	0.24

## Discussion

Craniofacial development progresses in an organized manner, varying in rhythm and magnitude due to heredity, gender, and ethnicity. It is this variability that adds complexity to our understanding of facial growth, necessitating a nuanced approach in orthodontic treatment. Understanding growth timing, magnitude, and direction helps orthodontists optimize treatment. Khadilkar et al. identified peak height velocity at 10.5 years in girls and 13.5 years in boys for Indian children [6]. Building upon this foundation, this longitudinal study was conducted to assess changes in facial traits for girls and boys, and then compare these changes between girls and boys in relation to an increase in standing height.

Table 2 highlights ANB angle reduction, attributed to differential maxillary and mandibular growth. According to Hamamci et al. [13], vertical facial growth is known to be related to skeletal maturation and somatic growth, which contribute to the increase in upper, lower, and total anterior facial heights; the present findings are consistent with previous research. Downs [14] and Bjork [15] observed greater vertical ramus growth than facial profile growth, causing forward-upward mandibular rotation and a reduction in mandibular plane angle. B-Pg and Go-Me were constructed to measure the change in the symphyseal region, which had reduced. This rotation indicates a change in inclination of the mandible relative to the anterior cranial base. The reduction in the angle Go-Gn to SN signifies an upward and forward rotation of the mandible. Jaw angle remodeling reduces the visible impact of mandibular rotation on craniofacial structure [16]. The changes in hard tissue parameters are in accordance with results obtained by Nanda et al. [17,18], Bishara et al. [3,19,20], Thilander et al. [16], and Hamamci et al. [13] (Figures 6, 7).

Table 2 shows soft tissue changes. Subtelny first documented nasal growth extending downward and forward with maturation [21]. The human nose undergoes continued growth, extending downward and forward at least until early adulthood [21,22]. Nasal projection and lip dimensions increased, aligning with Nanda et al. [18], Bergman et al. [22], and Mamandras [23]. The facial profile angle in this study was reduced. In a longitudinal study conducted by Bergman et al. [22], the facial profile angle decreased until the age of 12 in females, before rising again by 18 years, although not statistically significant, while in males, the angle decreased until the age of 14, before rising again by 18 years. This contrasts with Subtelny's [21] findings that the soft tissue profile convexity minimally changed from 6 months to 18 years.

Wisth's [24] study noted a decrease in skeletal facial convexity in both genders, while soft tissue facial convexity, excluding the nose, remained stable. Nasolabial angle in this longitudinal study was reduced. Hamamci et al. [13] mentioned in their study that the change could be attributed to the fact that the nasal septum and ANS provide support to the tip of the nose. As individuals age, the ANS moves forward, causing point A to shift relatively further away over time. With the lowering of the nasal tip, the nasolabial angle becomes more acute. As the nasal tip descends and rotates, the lip descends with it in what is called a clockwise rotation of the nasolabial complex [25]. With continuing growth, the chin tends to assume a forward position relative to the superior aspects of the skeletal face, and the mandible grows from the more retruded to a less retruded position [26] (Figures 8, 9).

Table 3 presents gender-based comparisons using an unpaired t-test. ANB, Go-Gn to SN, gonial angle, and B-Pg to Go-Me angle were reduced in both genders. Whereas upper anterior facial height, lower anterior facial height, effective midfacial length, ANS-PNS (maxillary length), effective mandibular length, and Go-Pg distance increased in both girls and boys. Out of these parameters, the overall increase in upper anterior facial height and effective mandibular length showed a statistically significant difference and was greater in boys as compared to girls. Facial profile and nasolabial angles decreased in both genders, while nasal projection, upper/lower lip thickness, upper lip length, lower lip to chin length, lower face to throat angle, and soft tissue chin thickness increased in both groups. Out of these parameters, the overall increase in soft tissue chin thickness showed a statistically significant difference and was greater in boys as compared to girls.

Over T2-T0, height increased by 6.6 ± 4.0 cm in girls and 6.9 ± 2.1 cm in boys, aligning with Goyal and Khadgawat [27], who reported peak height velocity at 10.5 years (6.6 cm/year) in girls and 13.5 years (6.8 cm/year) in boys. Nanda et al.'s research demonstrated that the soft tissue thickness over the chin, symphysis thickness, and length of the mandibular corpus all increased with age, with males exhibiting the most significant growth [18]. Nanda [17] reported that up to the age of 7, the size of the mandibular corpus was consistent between sexes, with both following parallel growth curves until age 15. After this age, males exhibited larger increases than females. This may explain why, in boys under 15, both sexes showed an overall increase of approximately 4.3 mm, with males likely to experience greater, prolonged changes.

Mamandras [23] reported vertical lip growth completion at 14 years in females, while in males, it plateaued at 18 years. Mandibular lip length increased until 16 in females but remained incomplete in males at 18. Lip thickness in females thickened until 14, maintained consistency until 18, then began thinning. Males reached maximum thickness by 16, followed by thinning. Nanda et al. [18] found that lip thickness uniformly increased from 7 to 18 years. Females achieved full thickness by 13, with slight thinning thereafter, while males continued thickening until 18. This suggests a cephalocaudal gradient of growth, with girls showing earlier lip growth than boys, aligning with soft tissue and skeletal growth patterns.

Table 4 shows the correlation between standing height and hard/soft tissue parameters across T1-T0, T2-T1, and T2-T0 in both genders. In girls, the effective mandibular length, lower anterior facial height, B-Pg to Go-Me, lower lip to chin length, and soft tissue chin thickness displayed a significant positive correlation with the incremental increase in standing height during T1-T0, while the soft tissue chin thickness continued to have an overall significant positive correlation during the total time frame (T2-T0). Therefore, in girls the increase in hard tissue parameters, specifically the lower anterior facial height and effective mandibular length, and reduction in B-Pg to Go-Me demonstrated a notable correlation with the increase in standing height during the time frame T1-T0, which might indicate a spurt, which was closely trailed by the augmentation in lower lip to chin length, and the thickness of soft tissue chin.

In boys, lower lip to chin length had a significant correlation during T1-T0 and ANB during T2-T1, but was not significant during the total time frame (T2-T0). Whereas the upper anterior facial height and ANS-PNS (maxillary length) showed an overall change, which displayed a significant positive correlation with the incremental increase in standing height in boys during the total time frame (T2-T0). Changes in boys were evenly distributed across time frames.

A limitation of this study is the sample size, which, while sufficient for identifying significant trends, may not fully represent the variability in craniofacial growth patterns across a larger population. Additionally, the use of lateral cephalograms provides valuable insights into profile changes but does not capture the full three-dimensional complexity of facial growth. Future research incorporating larger cohorts and advanced imaging techniques could further enhance the understanding of these developmental changes.

## Conclusions

The study findings indicate that in both genders, skeletal parameters such as effective midfacial length, ANS-PNS (maxillary length), effective mandibular length, and upper and lower anterior facial heights showed an overall increase. Conversely, ANB, mandibular plane angle, gonial angle, and symphyseal inclination exhibited a decrease. Regarding soft tissue changes, there was an increase in nasal projection, upper and lower lip dimensions, and chin thickness, whereas reductions were observed in the facial profile, nasolabial angle, and lower face-throat angle. Notably, boys demonstrated a significantly greater increase in upper anterior facial height, effective mandibular length, and chin thickness compared to girls. Additionally, significant correlations were identified between standing height and various craniofacial parameters. In girls, standing height was significantly associated with lower anterior facial height, effective mandibular length, symphyseal inclination, lower lip to chin length, and soft tissue chin thickness. In boys, standing height showed significant correlations with upper anterior facial height, ANS-PNS (maxillary length), and lower lip to chin length. These findings highlight the differential growth patterns and their relationship with overall stature in both genders.
